# Perspectives on Technology-Assisted Relaxation Approaches to Support Mind-Body Skills Practice in Children and Teens: Clinical Experience and Commentary

**DOI:** 10.3390/children4040020

**Published:** 2017-04-04

**Authors:** Timothy Culbert

**Affiliations:** PrairieCare Medical Group, Chaska, MN 55419, USA; tculbert@prairie-care.com; Tel.: +1-952-903-1380

**Keywords:** technology, mind-body skills, biofeedback, mobile applications, meditation, multimedia, computer games, children, relaxation, stress, video games

## Abstract

It has been well-established that a variety of mind-body (MB) techniques, including yoga, mental imagery, hypnosis, biofeedback, and meditation, are effective at addressing symptoms such as pain, anxiety, nausea, and insomnia, as well as helping with a wide variety of medical, emotional, and behavioral issues in pediatric populations. In addition, MB skills can also be health-promoting in the long-term, and with regular practice, could potentially contribute to longer attention spans, social skills, emotional regulation, and enhanced immune system functioning. Importantly, the benefits accrued from MB skills are largely dose-dependent, meaning that individuals who practice with some consistency tend to benefit the most, both in the short- and long-term. However, clinical experience suggests that for busy patients, the regular practice of MB skills can be challenging and treatment adherence commonly becomes an issue. This commentary reviews the concept of technology-assisted relaxation as an engaging and effective option to enhance treatment adherence (i.e., daily practice) for pediatric patients, for whom MB skills have been recommended to address physical and mental health challenges.

## 1. Introduction

Interactive electronic devices can play a positive role in healthcare [1,2,3,4,5,6,7,8,9,10,11,12,13,14,15,16,17]. Technology-assisted relaxation is currently a very popular topic for healthcare consumers, including products which are available in the form of multimedia mobile applications for smartphones or tablets. Devices can be used in a variety of situations and are available in numerous formats, such as teaching relaxation techniques, providing interactive electronic physiological monitoring (biofeedback) which facilitates self-regulation, or stand-alone electrotherapeutic technologies, which can influence brainwave frequencies. Therapeutic technologies are rapidly emerging as a unique and effective way to provide health information and as a source for patient self-management tools. Recently, a number of new options for mind-body (MB) skills training have been developed, including technologies that support stress management approaches, emotional regulation strategies, and self-calming techniques. Although many healthcare providers are aware of the need for effective stress management strategies for patients of all ages, busy clinicians may not have the time to stay up-to-date with these new technological offerings. However, in many cases, companies are launching direct-to-consumer advertising of their product benefits, which allows patients and families direct access to, and use of, these approaches, as they seek attractive options for treating stress-mediated mental and physical challenges.

It is well-known that children are regularly utilizing interactive electronic media, such as smartphones or tablets, at home and school [18]. In a 2015 article in *Pediatrics* by Kabali et al. [19], the following was discovered about the use of mobile devices by children and families: “[a] cross-sectional study [was performed including] 350 children aged 6 months to 4 years, seen October to November 2014, at a pediatric clinic in an urban, low-income, minority community. The survey was adapted from Common Sense Media’s 2013 nationwide survey … [and it was found that] most households had television (97%), tablets (83%), and smartphones (77%). At age 4, half the children had their own television and three-fourths [had] their own mobile device. Almost all children (96.6%) used mobile devices, and most started using before one years of age. Parents gave children devices when doing house chores (70%), to keep them calm (65%), and at bedtime (29%).” This study suggests that, even if not explicitly stated, many parents are currently utilizing technology with their children as a non-pharmacological option for purposes of calming and distraction.

After over twenty years working in the clinical practice of developmental/behavioral and integrative pediatrics, the author has witnessed the many benefits of teaching children a variety of MB skills, as an important therapeutic element in a comprehensive treatment plan. This commentary offers his opinions and perspectives on the way in which technology can enhance the process of learning and practicing MB skills for children and teens. Therefore, this paper is not intended to provide an exhaustive review of the literature on this subject, but instead is meant to reflect the author`s clinical experience and what he has found to be useful in his own clinical practice.

One important goal of this commentary is to raise awareness of these technologies among clinicians, so that healthcare providers might better understand alternative therapeutic options and consequently discuss these options with their patients. An additional hope is that healthcare providers may creatively integrate these newer technology-assisted relaxation approaches, when indicated, into appealing treatment strategies for pediatric patients for whom MB skills are indicated.

### 1.1. Complementary Approaches and Pediatric Practice

Complementary and alternative medicine (CAM) is quite popular for children and teenagers seeking natural approaches [20,21,22] for a variety of medical and mental health issues, partly because families are now seeking out more natural and less invasive therapies. Many children and parents prefer non-pharmacological therapies, if available, for common complaints such as headaches, abdominal pain, insomnia, anxiety, stress, inattention, anger, or emotional dysregulation. Within the CAM domain, MB and self-regulation skills are particularly effective in managing a number of common symptoms of childhood with a very positive risk/benefit ratio in a safe, cost-effective, and time-efficient manner [23]. The attractiveness of these options can be seen in a recent review by Vohra and McClafferty et al., who note that MB therapies “are popular and ranked among the top ten complementary and integrative medicine practices reportedly used by adults and children” [1].

### 1.2. Chronic Over-Arousal

It is also reasonable to argue that, for most children and teenagers, the demands and exposures of modern life are increasingly leading to chronic autonomic nervous system (ANS) over-arousal, in a way that is often unrecognized by the majority of individuals, who commonly accept it as their normal baseline reference mode of experience. However, this constant over-arousal can cause physical and emotional regulation problems [24,25,26]. In fact, since this state of ANS imbalance (stress) is so often recognized as a normal baseline mood, individuals may feel uncomfortable with calmer, quieter states of mind and body, as these can feel unfamiliar or uncomfortable. Evidence suggests that this heightened baseline and continuous state of chronic stress may be depleting for the mind, body, and spirit, and when left unchecked, can lead to many physical and mental health problems [27]. For example, stress may serve as an epigenetic modifier [28,29], while accessing a relaxation response achieved from practicing MB techniques (i.e., dialing up parasympathetic nervous system activity and reducing sympathetic arousal), may counterbalance this stress-effect and help to turn on health-promoting genes and processes.

A chronic state of nervous system over-arousal can also contribute to the etiology and/or maintenance of a number of physical issues (inflammation, pain, immune dysregulation, insomnia) and mental health symptoms (anxiety, depression, inattention), for which medications are commonly prescribed [24,25,26,27]. For these reasons, it is important that all children and teenagers learn MB balancing skills early on, so that they might also have the option to utilize non-pharmacological alternatives that can facilitate the MB`s natural healing capacity and perhaps address the causative issue at a more foundational level. It has also been well-established that the regular practice of a variety of MB techniques, including yoga, mental imagery, hypnosis, biofeedback, and meditation, are effective for re-balancing the ANS in pediatric populations and for addressing physical and emotional symptoms, such as pain, anxiety, insomnia, and gastrointestinal complaints [1,30,31,32]. Additionally, with regular practice, MB skills can be health-promoting in the long-term, contributing to better attention spans, social skills, emotional regulation, enhanced immune system function, and decreased inflammation [6,25,29,33].

### 1.3. Mind-Body Skills for Autonomic Nervous System Re-Balancing

Currently, there are no definitive studies detailing the exact amount of practice of MB skills required for optimal success, in terms of short-term symptom control (e.g., to reduce acute pain or anxiety), or long-term health and wellness improvements (e.g., baseline levels of stress, happiness, attention, inflammation). In clinical settings, some individuals do appear to achieve benefits—at least acutely—with only a single practice or a few experiences, but mastery and sustained change likely requires consistent practice of the skill, in order to use these skills effectively for the re-patterning of neurobiological processes and consequently create a new baseline. Therefore, it can be reasonably assumed that the benefits accrued from MB skills are likely dose-dependent. Despite the recognition that cost-efficient, safe, and effective MB techniques are readily available and can be used to address a variety of the aforementioned issues, children and teenagers often don’t practice MB skills, and certainly not to an optimal level to be able to achieve the best possible therapeutic benefit.

Clinical experience suggests that: (1) although many kids are familiar with the concept of relaxation, few have learned any formal MB skills; (2) many kids, when asked how they relax, suggest alternative methods, such as to read a book, do an art project, play a video game, or watch TV, which may or may not be objectively relaxing in terms of ANS arousal; (3) kids that have learned MB skills don’t practice them consistently in the appropriate situations; (4) kids that practice MB skills often get bored with that practice; (5) kids that understand and have practiced some form of MB skills are not necessarily applying them in a way that could be effective in-the moment; and (6) kids often don’t believe that a change in their thinking and feeling can really result in a physical change in the body.

### 1.4. Technology for Healing

The selective use of technology, although problematic in many ways, can be quite useful for children and teenagers to learn and practice MB skills, especially for those who have been reluctant to do so because it is perceived as another boring, non-preferred task, such as homework or household chores. Most individuals would prefer to interact with electronic media in the age of cellphones, computers, video game systems, or TV sets. This preference can be used to the advantage of healthcare providers, by becoming familiar with new “healing technologies”, that can be harnessed to provide technology-assisted relaxation training and can make the process of learning and practicing effective MB skills much more engaging, playful, and user-friendly for kids.

Many studies have focused on the potential downside of technology, suggesting that too much exposure to electronic media and/or exposure to certain types of inappropriate electronic media content, may have negative impacts on the developing child, such as contributing to a short attention span, sedentary behavior, over-arousal, aggressive behavior, depression, insomnia, and unhealthy food choices related to advertisements. However, not enough attention has been paid to the concept of utilizing these interactive electronic multimedia devices in order to engage children and teenagers in constructive, health-promoting, self-care skill development and symptom management. In this commentary, concepts such as play, therapeutic play, self-regulation, and MB skills, and their relationship to new electronic media, are reviewed. This commentary also reviews some of the most helpful software and devices currently used in the field of technology-assisted relaxation.

## 2. Benefits of MB Skills

MB techniques typically focus on the interactions and connections between the brain, the body, and the subsequent behaviors. There are many ways in which emotional, spiritual, cognitive, and psychosocial phenomena can directly affect physical and mental health. A compilation of potential benefits of practicing MB skills is listed in Table 1.

Teaching self-regulation and/or MB skills of any kind often involves assisting individuals to achieve a state of improved MB health and healing by shifting the balance of: (1) the ANS (from sympathetic activity to parasympathetic activity); (2) emotions (from negative to positive); (3) thoughts (from unhelpful to helpful) and; (4) a sense of control over health status (from external to internal) [23,33].

## 3. The Interface of Video Games, Self-Regulation and Healing

Many healthcare providers know that, when teaching kids self-care skills, they are more effective and more likely to be practiced and operationalized, if the skills are customized to the child’s interests and learning style, and delivered in a way that is playful, non-threatening, developmentally appropriate, and novel. Delivering treatments through “play” experiences or games, is an ideal, developmentally appropriate way to proceed in therapeutic interaction, and is often referred to as “therapeutic play.” Over the past several years, playing video games, texting, and watching online videos have become popular activities for children and teens [34,35,36]. What exactly is it that seems to make video games so popular? One component of their appeal may involve the experience of “mastery” and being recognized as an expert. For a child with limited opportunities to experience success in activities such as sports, music, or theater, being talented at video games could have a positive impact on their self-esteem.

Additionally, video game designers also know what it takes to engage kids. In their 1983 book *Mind at Play: The Psychology of Video Games*, psychologists Loftus and Loftus describe the three elements that drive the design of successful, addictive video games and make them intrinsically motivating (i.e., curiosity, fantasy, and challenge) [37]. These concepts are still relevant and can therefore be used to inform the design of effective MB, self-care strategies for kids. In particular, children aged four to 13 years are developmentally driven towards mastery, have a rich fantasy life, move easily between real life and their imagination, and are engaged by experiences that are multimedia, interactive, and novel—thus evoking a strong sense of curiosity. The designers and marketers of computer games, mobile apps, and related software and gadgets have often exploited these elements in the design of popular multimedia entertainment that have become highly recognized brand names, such as *Final Fantasy*, *Angry Birds*, *Legend of Zelda,* and *Kingdom Hearts*, along with many games based on movies and cartoons, such as *Toy Story*, *Pokemon* and *Cars*. Interestingly, popular children’s books are now among the top-sellers for mobile apps, but have also gone far beyond the book form—interactive elements have been added, such as gaming features and audio enhancements (i.e., entertaining sound effects).

Consequently, technology-assisted relaxation devices have been increasingly designed to harness the same power, absorption, and fun of these electronic games features and mobile applications, but for the service of health instead of strictly entertainment. Multimedia interactive game play can be utilized to connect with the imagistic, emotional, and sensory elements of the right brain for symptom management and healing. The following section will review the concept of MB skills, along with current electronics–based options for kids and teenagers that leverage the idea of “therapeutic play”, in order to practice MB skills at home, at school, or in clinical settings. This listing is not complete, but has been gathered by the experience of the author, who has personally used the majority of these products in both inpatient and outpatient settings. Additionally, the paper will review common products that the author and his colleagues have found useful for supporting children in their regular, daily practice of MB skills. These products are organized by the type of technology involved: (1) mobile applications (for smart phones and tablet computers), which only involve software; (2) biofeedback devices (either stand-alone or computer-based), which involve hardware (a physiological sensor) and software; (3) other technology-assisted relaxation devices that are specialized portable technologies involving electrical stimulation.

## 4. Definitions of MB Skills

1. *Breathwork:* the conscious control of the rate, rhythm, and depth of breathing; a technique used in relaxation and meditative practices in order to reduce psychological stress and increase parasympathetic nervous system activity.
2. *Biofeedback*: a technique you can use to learn to control your body’s functions, such as your heart rate, hand temperature, or muscle tension. With biofeedback, you’re connected to electrical sensors or electromechanical devices that provide you with auditory and visual information about your body. This feedback helps you to then control a desired physiological function in a therapeutic direction, which provides the opportunity to more objectively observe the mind-body connection and understand how a change in thinking or feeling can directly correlate with a change in physiology.
3. *Guided and/or Mental Imagery/Self-hypnosis*: commonly invokes all of the five senses in journeys of imagination (and fantasy) and facilitates an intensification of focus, less attention to peripheral factors, more awareness of internal events/sensations, and enhanced suggestibility.
4. *Meditation and Mindfulness*: practices involving concentrated focus or intentional attention training, in order to increase awareness of the present moment or to reduce stress.
5. *Yoga*: a Hindu spiritual and ascetic discipline derived from the Sanskrit word *yuj*, meaning union. It includes body postures, breathwork, and meditative practices.

## 5. Electronic Mobile Applications for Smartphones and Tablet Computers

Mobile apps are often game-like in design and can include features of immersive, attention-maintaining properties of fantasy and imagery, offer interactivity and control, and can include useful behavioral change elements, such as symptom assessment, tailored messages, tracking progress, and goal setting.

### 5.1. Mobile Apps for Breathwork

#### 5.1.1. Breathe2Relax

According to its website [38], “this (app) is a portable stress management tool which provides detailed information on the effects of stress on the body and instructions and practice exercises to help users learn the stress management skill called diaphragmatic breathing.” This app also includes effective explanations and illustrations of the respiratory system.

#### 5.1.2. Breathing Zone

This is a well-designed mobile app that basically serves as an interactive breath pacer. The user has the flexibility to pick the beginning breath pace, ending breath pace, and cueing audio for each breath (chime, water, voice, etc.), along with an optional timer.

#### 5.1.3. Belly Bio Interactive Breathing

This is an interactive app for the iPhone that utilizes the phone’s accelerometer to monitor proper diaphragmatic (belly) breathing techniques, by providing feedback about the movement of the user’s stomach.

#### 5.1.4. Breathing Bubbles

*Breathing Bubbles* is an app that helps kids to practice releasing worries and focus on pleasant feelings, while using a bubble as a visual representation for this release.

### 5.2. Mobile Apps for Meditation and Mindfulness

Meditation has been proven to be a helpful tool addressing medical and mental health issues, for both the pediatric and adult populations [2,31].

#### 5.2.1. Take a Chill

According to the developers [39], “this mobile app offers mindfulness meditation exercises and texted practice reminders. As a teen, there are moments in your life when you’re really stressed and just need to ‘Take a Chill’.” This app is full of tools to help manage that stress and incorporate mindful practices into a daily routine. Using quick mindful exercises and thoughtful activities, teenagers learn to begin to overcome those moments, whether it’s studying for a test or preventing negative thoughts and patterns. Features in the app include:

1. Quick Exercises: two illustrated exercises for in-the-moment situations
2. Daily Dose: daily activities to help reinforce concepts and increase your mindful practice
3. Progress: see your progress and activity overtime
4. Reminders: keep yourself motivated with three types of Reminders
5. Motivational Quotes: receive a new motivational quote after each use

#### 5.2.2. Stop, Breathe and Think

The creators [40] state that, “with this app, you can develop and apply awareness, kindness and compassion in your daily life through a meditative process called *Stop, Breathe and Think*: (1) Stop: Stop what you are doing. Check in with what you are thinking, and how you are feeling; (2) Breathe: Practice mindful breathing to create space between your thoughts, emotions and reactions; (3) Think: Learn to broaden your perspective and strengthen your force field of peace and calm by practicing one of the meditations.”

#### 5.2.3. Smiling Mind

The creators of this app [41] state that, “*Smiling Mind* is a modern meditation approach, a unique web- and app-based program developed by psychologists and educators to help bring balance to people’s lives by teaching meditation and related skills.”

### 5.3. Mobile Apps for Yoga

Yoga can be used as another helpful tool to address a variety of psychological and physical conditions of childhood [30].

#### 5.3.1. The Adventures of Superstretch

This is a mobile app [42] that teaches yoga with videos of real kids doing yoga postures, and animated narrators offering information, instructions, and insight. It is a helpful way to get sedentary kids moving in a fun, non-threatening process. The app’s designers have declared that “kids don’t have to be athletes, flexible or fit to do this. We’ve designed it to be non-competitive and even bring in partner activities to show we’re all in this together. In yoga, there’s no perfect. In each pose, the kids are just being kids—doing poses the best they can. No need to be perfect, to ‘succeed’ or do it ‘right’. We are allowing ourselves to be present, in the moment. It’s ok to fall out of poses. It’s ok to laugh, to roar like a lion—in fact, that’s the whole point. Be courageous, creative and just try.”

#### 5.3.2. Yoga by Teens

According to the developers [43], “within this app, kids can join yoga teens, Lauren, Emily and Jessica Parsons in a class that is designed and taught by teens. *Yoga by Teens* teaches basic hatha yoga poses that are fun, challenging and help to build strength and flexibility, as well as develop self-confidence and self-esteem. Its message is that yoga is for all ages and levels of ability, including people with special needs.”

#### 5.3.3. Kids Yogaverse: I Am Love

The website [44] claims, “this is a great app which allows kids to calm down and be ready for the day. It teaches individuals to always think about good things and always know that you are smart. It is beautifully hand-drawn and incorporates 13 yoga poses for children demonstrated by children. Get ready to open your heart, expand your mind and reveal that *you* are the real treasure to love. The yoga poses help you to stretch out and wake up your mind. The material on this app can be enjoyed by listening to the book, or reading the book.” Additional interactive features include:

1. Animated Poses: follow the children as they do the real poses through the yoga journey
2. Breath Affirmations: press the Breath button on each page and you will hear a special message just for you
3. Guess What?: press the Guess What button and learn amazing new facts

### 5.4. Mobile Apps for Imagery, Movement and More

#### 5.4.1. Healing Buddies Comfort Kit

The author has developed this free mobile app with the local children’s hospital, called the *The Healing Buddies Comfort Kit,* for use in children’s hospitals and clinical settings. It provides an interactive, game-like, colorful, multimedia way for kids and teens to learn a variety of integrative and holistic techniques in order to manage bothersome symptoms. It includes guided sessions of imagery, muscle relaxation, and breathing for a variety of symptoms that kids might experience at home, in hospitals, or anywhere, such as pain, nausea, fatigue, insomnia, and anxiety.

#### 5.4.2. Me Moves

This app (now available as a digital download) is accessible on tablet computers, cellphones, and computers. The interaction involves using your arms and upper body to slowly follow and engage in graceful upper body movements, to the beat of a metronome and lovely background music. Kids with any kind of over-arousal or anxiety tendencies, including kids with autism and ADHD, can greatly benefit from this program. The developers [45] state that, “mirroring those on the screen, users are transformed by the music, images, movement, and the expressive features of emotion. This easy to use, interactive program has been widely praised by therapists, educators, and parents.”

## 6. Biofeedback Devices (Stand-Alone, Computer-Based and Smartphone-Based)

Training MB self-regulation skills using biofeedback-based relaxation techniques and technologies can be quite engaging, interactive, and effective for treating a variety of conditions [1,3], which has been described by kids as “a videogame for your body”. A therapist might use several different biofeedback methods. Determining the method that is right for your client depends on their specific health problems and goals. Biofeedback methods include:

1. *Brainwave*: also known as an electroencephalograph (EEG) and neurofeedback, this type of device uses head sensors to monitor brain wave frequencies.
2. *Heart rate*: this type of biofeedback uses an infrared finger or earlobe sensor, called a photoplethysmograph, to measure your heart rate and variability (HRV).
3. *Muscle*: electromyography (EMG) biofeedback, which involves placing sensors on skeletal muscles to measure the electrical activity that causes muscle contraction.
4. *Sweat glands*: sensors attached around your fingers or on your palm measure sweat gland activity (also called electrodermal activity; EDA).
5. *Temperature:* sensors read the temperature of your fingers/hands that is controlled by blood flow to the periphery (which is influenced by ANS balance).

### 6.1. HeartMath Products (Heart Rate Variability)

These products are available as a Mac- or PC-based program with game features (*emWave Pro* and *emWave Pro Plus*), as a mobile app for smartphones or tablet computers (called *Inner Balance*), or as a portable, handheld version (called *emWave2*, which features color changing LEDs that signal the user about ANS balance and an optimal state of psychophysiological harmony, termed “coherence”.) The user receives information about the heart rate variability pattern as a proxy for ANS balance and emotional regulation. *HeartMath’s emWave Pro* also contains video games, for example one game challenges the user to color in a picture that starts as black and white as they move into a more optimal ANS balance and shift to a positive emotional state. Another game format rewards the user with a hot air balloon ride over beautiful landscapes. With the newest game, dual drive, the user controls the speed and handling of a sports car by staying in optimal ANS balance. With the portable, handheld version (*emWave2*), the user can follow a breathing pacer and will then see a specific LED change from red (low coherence), to blue (medium coherence), to green (high coherence). Features of “Inner Balance” include the ability to customize screens with photographs and personally-selected music. This serves as a great tool for kids with any kind of chronic pain, anxiety, insomnia, tics, or anger management challenges.

### 6.2. Muse (Electrical Brain Activity)

The Muse is a brain-sensing headband that measures electrical brain signals using seven finely calibrated sensors—two on the forehead, two behind the ears, and three reference sensors. The headband uses information from these EEG sensors to measure whether your mind is calm and focused, or active and wandering, and translates that data into active sounds to guide you. There is a companion app for smartphones or tablets that provides audio and visual feedback to guide training.

### 6.3. The Pip (ElectroDermal Response)

The skin pores (sweat glands) on your fingertips are sensitive to changing levels of stress. *The Pip* accurately captures these changes, and through audio and visual feedback, allows you to be aware and change your response pattern. It achieves this by detecting variations in your EDA. This product comes with interactive software to provide audio and visual feedback and summary information about stress response patterns.

### 6.4. eSense (Peripheral Temperature Monitor)

According to its website [46], “*eSense* is a small sensor for measuring your skin temperature with the microphone input of your smartphone or tablet (Android or Apple iOS.). Hand warming training is an especially effective and common biofeedback method. The temperature of our hands is connected to our stress level: when we are stressed, we tend to have cold hands. Immediate feedback enables you to voluntarily increase the skin temperature in your fingers, thus increasing your peripheral blood circulation, leading to relaxation and stress reduction. *eSense* provides you, in various ways, with exact feedback about your momentary stress level (e.g., with measurement curves, video display and audio feedback features).”

### 6.5. Antense (Electromyography)

The developers [47] state that, “the *Antense* anti-tension device is a simple yet effective way to eliminate stress by helping you develop a greater sense of body awareness in the comfort and privacy of your home. A common cause of the discomfort associated with stress or tension is the contraction of your scalp, face and neck muscles. Learning how to relax these muscles is an essential step toward relief and prevention of this discomfort. Because these muscles are not normally under your conscious, direct control, a monitoring or guidance system is necessary to indicate or feedback to you their level of activity. EMG. The *Antense* biofeedback device provides this monitoring. The instrument continuously monitors and averages the EMG activity in the frontalis (forehead) muscle and converts it into a pleasant tone. Using patented technology, *Antense* measures these contractions and instantly converts the signal into a pleasant tone-pitch proportional to the level of muscle tension in your body. With the variations in the tone as a guide, you can quickly train your muscles to relax relieving the discomfort and preventing future problems related to tense facial muscles like headaches. By learning to feel how stress changes your body, you will quickly understand how to release that tension and stress and learn how to relax at will.”

## 7. Other Technology-Assisted Relaxation Devices

As technology marches on, there are some additional, very promising gadgets that can assist individuals in achieving a deep relaxation response and decrease sympathetic nervous arousal, while enhancing parasympathetic nervous system activity. The available scientific literature regarding the use of these devices by children and teens is very limited, so they should be used with caution. However, these devices are generally safe and easy to use, and are available for anyone to purchase, without the prescription of a healthcare provider. Although there is still only limited research on the use of these technologies, they are worth considering and have shown promising preliminary results in adult populations [48,49,50].

### 7.1. Cranial Electrotherapeutic Stimulation (CES)

The concept of applying electrical stimulation for healing purposes has been in use for at least 2000 years. Machines that deliver a small, pulsed electric current across the head are now claiming efficacy for a variety of medical and mental health conditions. Cranial electrotherapeutic stimulation (CES) is a technology which applies small levels of electrical stimulation across the cranium, to benefit patients with issues such as anxiety, depression, insomnia, ADHD, and chronic pain [41,43,44].

One of the developers of a CES product called *alpha-stim* [51], states that, “our brain basically creates four different frequencies of brain waves each day: beta, alpha, theta and delta. Alpha waves are created when we are in a state of quiet, calm, focused contemplation, and are often associated with meditative states and practices. The CES device sends pulses of very low amperage (i.e., less than 1.0 milliampere) electricity through thin wires attached to electrodes clipped to the ear lobes or stuck to the skin over the bony prominences just to the front of, or behind each ear. The frequency of the electrical pulses can be adjusted—usually from 0.5 Hz to 100 Hz—depending on the treatment effect desired.” You could think of these new gadgets that passively cultivate alpha waves as a passive form of meditation. Basically, you place small sensors on your earlobes, dial up the device (about the size of a thick cellphone), and passively enjoy the cultivation of alpha brain waves. Although the exact mechanisms of alpha wave stimulation are not known, some have claimed that these waves could promote the release of neurotransmitters, like serotonin, and the elicitation of alpha wave frequencies in the brain. In alpha wave states, people tend to feel calm, relaxed, and report an enhanced mental focus. Products include *alpha-stim*, *CES Ultra*, and *Fisher Wallace* (Table 2).

### 7.2. Audio-Visual Entertainment (AVE)

The brain is capable of being tuned in a remarkable variety of ways. For instance, certain music or light-pulsing colored sunglasses can significantly alter brain wave frequency, which affects different functions, depending upon the chosen frequency. Beta waves are associated with active problem solving, alpha waves with a calm, focused mind, theta with creative thinking, and delta waves with sleep. Brainwave entrainment refers to any practice intended to cause brain waves to fall into step—or certain frequencies—by using a periodic stimulus of some type. One potential desirable result of brainwave entrainment is to synchronize the two hemispheres of our brain with their brainwave frequency patterns. Potential benefits of hemisphere synchronization include promoting happiness, optimism, and contentment [52,53,54].

For simple audio entrainment, products include various music or nature sound CD’s with embedded beats, vibration, and or therapeutic suggestions. Examples of this include CD’s by Jeffrey Thompson, and holo-synch and hemi-synch recordings. When pulsing LED lights are added to audio entrainment, audio-visual entrainment is subsequently created: products include the *DAVID*, the *Dream Machine*, and *Proteus* (Table 2). Some of the benefits which have been seen from the use of brain entrainment include: healthier sleep patterns and more restful sleep each night; a greater ability to relax and meditate (some describe brain entrainment as the ultimate meditation tool); a lowering of situation-specific and overall stress levels; improvements in cognitive function and creativity; easing of negative emotions which affect health and happiness; and an overall sense of peace and well-being.

### 7.3. Transcutaneous (tc) Vagal Nerve Stimulation (tc-VNS)

The concept of influencing neurons with electricity has been used for over a century, when patients were treated with electro convulsive therapy as a treatment for severe depression. Additionally, and more recently, transcranial electromagnetic stimulation has also been gaining traction as a treatment for insomnia and other conditions [55,56]. The vagus nerve originates in the brain and vagal nerve stimulators electronically signal the vagus nerve (more specifically, they stimulate the afferent auricular branch of the vagus), which in turn stimulates the release of neurotransmitters in the brain, in order to generate a calming sensation. Stimulation of the vagal nerve also increases parasympathetic nervous system activity, which can potentially elicit a relaxation response. This can occur during the stimulation session, but also has longer lasting effects to decrease tension, if used daily. Surgically implantable vagal nerve stimulators are available and have been used to effectively treat seizures and depression. Wearable devices are now available that provide transcutaneous (tc) stimulation (and which do not require surgery). As of 2016, a device called *Nervana* offers an FDA-approved tc nerve stimulator, which combines music with vagal nerve stimulation in the ear canal, using ear buds.

## 8. Clinical Tips: Prescribing MB Skills Practice

One of the most important recommendations for prescribing the practice of MB skills to pediatric patients is to start small, such as a prescription for MB practice for only three to five minutes, once or twice a day. MB skills are best taught in an organized sequential manner, making certain that pediatric clients master each step along the way, as follows:

(1) Mind-Body Awareness
  - Learn to discriminate (objectively) the mind-body differences between sympathetic nervous system arousal (stress) and parasympathetic nervous system dominant states (relaxation response).
(2) Mind-Body Self-Regulation
  - Learn to modulate (first in a controlled environment) a given psychological or physiological function, in a therapeutic direction, consistently.
(3) Mind-Body Skill Transfer
  - Apply this ability to self-regulate the mind and body in appropriate life situations, as needed, “in the moment”.
(4) Mind-Body Reset
  - Restructure (CNS) and rebalance (baseline ANS) with regular daily practice to a calmer, happier, emotionally resilient pattern long-term.

It is also necessary to prescribe daily practice in two different modes; consistent daily practices to reset the neurological baseline, along with recommending an “in-the-moment”, brief application of techniques and technology, to manage acute situations in real life (e.g., feeling stressed, having a headache, trouble sleeping, getting angry). Have clients try and practice a variety of MB skills and gadgets so that they can figure out what works best for them. It is also ideal to have multiple options in one’s “self-care toolkit” and to request that parents provide rewards for regular practice and effort shown. Clients should also focus on keeping a symptom diary, and describe practice frequency, length, and daily situations they find challenging, stating whether any MB skills that they had tried were helpful. A summary of potential MB resources to recommend to patients is listed in Table 2.

## 9. Conclusions

The intention of this commentary was to provide an entertaining and informational perspective on the interface of technology, mind-body skills, and effective patient care for pediatric healthcare providers of all kinds. The reality is that computer games, interactive mobile apps, and other health-related gadgets are no longer just a trivial activity played by individuals for fun, but can now be utilized for various educational and therapeutic purposes. Consequently, the author intends to highlight the benefits of utilizing technology-assisted relaxation devices and strategies to enhance the practice of MB skills in children and youth in the service of healing and wellness promotion.

Well-designed computer games/apps have the potential to engage the player and are often designed to be intrinsically motivating, in order to encourage frequent use (the *Journey to Wild Divine* home biofeedback system serves as a good example of this) [57]. If technology-assisted relaxation devices and software provide positive reinforcement, mastery, and curiosity, kids and teens will be more willing to utilize them again, and therefore, therapeutic benefits can be gleaned from this approach. Although the use of technologies can be increasingly problematic in the life of children and teens, they can also be harnessed and adapted for therapeutic purposes. Technology-assisted relaxation can be quite useful (when carefully explained, prescribed, monitored, and utilized) as a culturally-syntonic vehicle to facilitate the regular practice of health-promoting MB therapies, such as breathwork, yoga, biofeedback-based relaxation, and mindfulness meditation. Therefore, healthcare providers should focus on understanding the latest technology available, and promoting self-regulation through MB skills training, which is known to provide benefits to humans of all ages.

In this age of rapid technological advancements, we could begin to imagine what the next frontier looks like: combining the best of the graphic design and multimedia arts, music, play, computerized hardware/software applications, and new miniaturized technologies for the creation of “therapeutic play” products, tools, and techniques for the next generation. Will the most effective therapists and pediatricians deliver their training on tablets and touch screen cellphones? Will clients check in regularly online via these devices? Will physiologic sensors be added into clothing or jewelry for additional information and options for more precise control of mind and body? As we continue to explore the interface of the arts, biomedical technologies, and healthcare endeavors in the service of healing, tremendous potential is available to develop new ways in which electronic media can enhance health and healing for kids and teenagers across a variety of settings, although vigilance is still needed to combat the potential downside to the overuse of technology [34].

Technology-assisted relaxation devices, in the form of computer games and electronic mobile apps, will certainly be part of the future of pediatric healthcare. Design features that optimize these healing technologies must take into account the specific client`s play style, audio and visual preferences, developmental stage, and attention span. These products should support patients in a variety of ways, including promoting health literacy and improving self-regulatory abilities, both physically and psychologically. From a personalized healthcare perspective, computerized games utilized to promote health and wellness can provide a new source of information about patient preferences, health status, psychophysiological response patterns, and self-care abilities. Healthcare providers will hopefully find creative ways to utilize technology in care planning, as a way to enhance treatment adherence with recommendations to practice MB skills, and ultimately, to improve outcomes for the children, teenagers, and families we serve.

## Figures and Tables

**Table 1:** Potential benefits of mind-body (MB) skills training.

Eliciting the “Relaxation Response”
Autonomic Nervous System Balancing: decreasing sympathetic nervous system activity while dialing up parasympathetic nervous system activity
Enhanced Emotional Regulation
Training Attention and Cultivating Awareness
Stress Management
Modifying the central nervous system (CNS)
Performance Enhancement
Improved Social Interactions
Improved Decision Making
Increasing Peace and Happiness
Building Emotional and Physical Resilience
Pain and Symptom Management

**Table 2:** List of MB resources.

Biofeedback	Other Technology-Assisted Relaxation Techniques	Mobile Apps
**HRV Biofeedback** www.heartmath.com	**AVE-Audio Entrainment** www.soundstrue.com www.centerpointe.com www.hemisynch.com	**Breathing** www.t2health.dcoe.mil www.breathing.zone www.bellybio.com
**EDA Biofeedback** www.thepip.com	**AVE-Audio Visual Entrainment** www.mindalive.com www.mindmachines.com www.deepakchopradreamweaver.com	**Me Moves** www.thinkingmoves.com
**EMG Biofeedback** www.lifematters.com	**CES** www.alpha-stim.com www.cesultra.com www.fisherwallace.com	**Healing Buddies Comfort Kit** www.healingbuddiescomfort.org
**TEMP Biofeedback** www.mindfield.de	**tc-VNS** www.experiencenervana.com www.cerbomed.com	**Meditation** www.buddhify.com www.stopbreathethink.org www.stressedteens.com www.smilingmind.com.au
**EEG Biofeedback** www.choosemuse.com		**Yoga** www.adventuresofsuperstretch.com www.kidsyogaverse.com
**MULTIMODAL BF** www.wilddivine.com		

HRV: heart rate and variability; AVE: audio-visual entertainment; EDA: electrodermal activity; CES: cranial electrotherapeutic stimulation; EMG: electromyography; tc-VNS: transcutaneous vagal nerve stimulation; TEMP: temperature; EEG: electroencephalograph; BF: biofeedback.
